# Dining-Out Behavior as a Proxy for the Superspreading Potential of SARS-CoV-2 Infections: Modeling Analysis

**DOI:** 10.2196/44251

**Published:** 2023-03-07

**Authors:** Ka Chun Chong, Kehang Li, Zihao Guo, Katherine Min Jia, Eman Yee Man Leung, Shi Zhao, Chi Tim Hung, Carrie Ho Kwan Yam, Tsz Yu Chow, Dong Dong, Huwen Wang, Yuchen Wei, Eng Kiong Yeoh

**Affiliations:** 1 Centre for Health Systems and Policy Research, Jockey Club School of Public Health and Primary Care The Chinese University of Hong Kong New Territories Hong Kong; 2 Center for Communicable Disease Dynamics, Department of Epidemiology Harvard T.H. Chan School of Public Health Boston, MA United States

**Keywords:** COVID-19, contact tracing, unlinked, superspreading, dispersion, public health, surveillance, digital health surveillance, digital surveillance, disease spread

## Abstract

**Background:**

While many studies evaluated the reliability of digital mobility metrics as a proxy of SARS-CoV-2 transmission potential, none examined the relationship between dining-out behavior and the superspreading potential of COVID-19.

**Objective:**

We employed the mobility proxy of dining out in eateries to examine this association in Hong Kong with COVID-19 outbreaks highly characterized by superspreading events.

**Methods:**

We retrieved the illness onset date and contact-tracing history of all laboratory-confirmed cases of COVID-19 from February 16, 2020, to April 30, 2021. We estimated the time-varying reproduction number (*R_t_*) and dispersion parameter (*k*), a measure of superspreading potential, and related them to the mobility proxy of dining out in eateries. We compared the relative contribution to the superspreading potential with other common proxies derived by Google LLC and Apple Inc.

**Results:**

A total of 6391 clusters involving 8375 cases were used in the estimation. A high correlation between dining-out mobility and superspreading potential was observed. Compared to other mobility proxies derived by Google and Apple, the mobility of dining-out behavior explained the highest variability of *k* (ΔR-sq=9.7%, 95% credible interval: 5.7% to 13.2%) and *R_t_* (ΔR-sq=15.7%, 95% credible interval: 13.6% to 17.7%).

**Conclusions:**

We demonstrated that there was a strong link between dining-out behaviors and the superspreading potential of COVID-19. The methodological innovation suggests a further development using digital mobility proxies of dining-out patterns to generate early warnings of superspreading events.

## Introduction

The COVID-19 pandemic has caused nearly 300 million confirmed cases and over 5 million attributable deaths worldwide during 2020-2021. As human mobility is a key driver of SARS-CoV-2 transmission [1], nonpharmaceutical interventions (NPI) aimed at reducing human movements and contacts, including travel restrictions, case detection, isolation, quarantine of close contacts, and social distancing, have been effective in flattening incidence curves and protecting the health care system from being overwhelmed [2]. Of the human activities, indoor dining at eateries exposes people to a high SARS-CoV-2 infection risk, especially since mask mandate does not apply in those occasions. Several investigations have shown that permitting on-premises dining was associated with increased risk of COVID-19 infections [3,4].

In the current pandemic, digital data encoding human mobility information have been highly leveraged for purposes of assessing risk of infectious disease transmission, quantifying the effectiveness and compliance of NPI, and enabling early detection early detection of cases to prompt timely interventions. To facilitate the control of the COVID-19 pandemic, several multinational companies with GPS-related services released their mobility data, such as the community mobility reports from Google LLC [5], mobility trends report from Apple Inc [6], and the COVID-19 mobility data network from Meta Data for Good program [7]. There are some more fine-grained digital proxies applied to construct the COVID-19 mobility networks, which entailed hourly movements to specific points of interest, including restaurants [8,9].

As a densely populated metropolitan, Hong Kong was vulnerable to considerable COVID-19 outbreak risk and has kept community transmissions at a containable level through case detection and isolation, intense contact tracing, and quarantine in the early phases of the pandemic [10,11]. However, the superspreading events (SSE), where a large number of secondary cases were generated by a few primary cases, presented challenges for the effectiveness of these measures. Previous studies indicated that approximately 20% of cases seeded 80% of all local transmissions in the first and second wave of COVID-19 epidemics [12,13]. Similar observations were also reported in other Asia-Pacific settings such as South Korea [14]. The government has rolled out different levels of measures to prevent transmissions by restricting capacity, limiting the number of diners per table, and shortening the dine-in hours. Eateries were found to be settings where major clusters with SSE occurred, even though corresponding restriction measures such as social distancing and limited dine-in hours were in place [15,16].

Many studies assessed the reliability of different digital mobility metrics as a proxy of the SARS-CoV-2 transmission [17-21]. For example, Kurita et al [19] demonstrated that Apple mobility data were useful for the short-term prediction of COVID-19 transmissibility. Nevertheless, to our knowledge, none have examined the relationship between the trend of dining-out behaviors and the superspreading potential of COVID-19. A good understanding of the relationship is essential for informing and evaluating the effectiveness of NPI. In this work, we aim to examine the association between the trend of dining-out behaviors and disease transmissibility in Hong Kong, a setting with COVID-19 outbreaks characterized by SSE.

## Methods

### Epidemiological Data

Surveillance data on local COVID-19 cases were provided by the Centre for Health Protection, Department of Health of the Government of the Hong Kong Special Administrative Region. All COVID-19 infections were confirmed by testing with polymerase chain reaction. We retrieved the illness onset date and contact-tracing history of the confirmed cases. Based on the contact history, we reconstructed the transmission clusters, defined as a number of cases with the same source of infection (ie, a primary case) or epidemiologically linked [22]. Cases without source cases that did not have epidemiological linkage with other confirmed cases were defined as sporadic cases (ie, cases with untraceable contacts). A sporadic case was considered as a cluster equal to one. The study period was from February 16, 2020, to April 30, 2021, when the third wave of transmissions was brought under control.

### Digital Mobility Proxy of Dining-Out Patterns

A web crawler was developed to retrieve user comments from OpenRice, the most commonly used restaurant catalog for people to search and provide feedback in Hong Kong. The website covers restaurant information and user comments for more than 28,000 eateries and is open for public access without a log-in requirement. We obtained the daily total number of comments as a proxy of dining out in eateries from February 16, 2020, to April 30, 2021. The daily counts were normalized using the baseline means of total comments between November 1 and December 31, 2018.

### Other Digital Mobility Proxies

We obtained anonymized and aggregated human mobility data released by Google and Apple during the COVID-19 pandemic by locations and transport modes [5,6]. The Google COVID-19 community mobility reports included the daily change of visitor numbers to 6 locations (ie, retail and recreation, grocery and pharmacy, parks, transit stations, workplaces, and residential) compared to the median value in baseline days (from January 3, 2020, to February 6, 2020). The commuting information was collected through GPS linked with the Google Maps app. Similarly, mobility data from Apple mobility trends were also obtained, which reports on the daily ratio of trip numbers to the baseline (January 13, 2020). The data sets were generated from users’ devices connected to the Apple Maps service for directions and were categorized by driving and walking.

### Estimation of the Time-Varying Reproduction Number

Following Cori et al [23], we estimated the time-varying reproduction number (*R_t_*) over the study period. In this approach, the daily number of cases by illness onset date was modeled through a branching process, where the incidence at time *t* (ie, *I_t_*) is Poisson distributed with the mean given by 
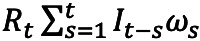
, where ω_*s*_ is the discretized probability distribution of the serial interval. Epidemiologically, a serial interval approximated the infectiousness of a COVID-19 case at time *s* after the symptom onset. We assumed the mean and SD of the serial interval during the study period to be 6.5 and 4.1 days, respectively, based on a previous study conducted in Hong Kong [24]. As the empirical serial interval estimates may be biased due to the sampling and selection bias within the contact-tracing data collected during an ongoing epidemic [25], we also tested another assumption of serial interval distribution (mean 4.6, SD 4.9 days) estimated from a modeling study that corrected for such bias [26] as a sensitivity analysis. We only considered cases that acquired the infection locally in the estimation. The *R_t_* was estimated by fitting the time-series data to the Poisson distributions in a sliding window process, assuming the *R_t_* remained constant within each window.

### Estimation of Superspreading Potential

Given the stochastic effect of transmissions, the transmission dynamics can be described by an offspring distribution (ie, the distribution of the number of secondary cases generated by the primary case). Following Lloyd et al [27], we assumed a negative-binomial offspring distribution, which was parametrized by a reproduction number and a dispersion parameter (*k*). When *k* is sufficiently low (ie, less than 1), SSE are more likely to occur. Applying the branching process theory, *k* could be estimated by fitting the transmission cluster data to a cluster size distribution [28], which describes the probability of clusters with a size of *z* seeded by *u* primary cases. We only included transmission clusters that were initiated by local cases. For an ongoing epidemic, the clusters’ sizes are likely to grow after the time of estimation. To this end, we also considered right censoring in the cluster size distributions [22,28,29]. Thus, the likelihood function is:



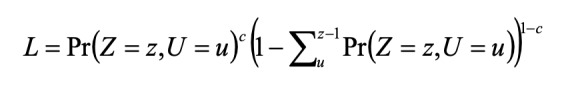



Here, Pr(*.*) is the probability mass function of the cluster size distribution assuming a negative binomial offspring distribution following previous studies [22]. The term *c* is an indicator of censoring, where *c*=0 if the cluster is censored and *c*=1 if the cluster is considered as self-limited. A cluster is censored if it has new confirmed cases within 11 days before the day of estimation [22]. We determined the time-varying *k* during the study period in a sliding window process.

The window length was fixed at 30 days, which could cover 4 transmission generations based on the upper bound of the estimated generation interval of COVID-19 [30]. Within each window period, we estimated *k* using the Markov chain Monte Carlo method. Gamma distribution and half-normal distribution were used as prior distributions for *R_t_* and *k*, respectively. For each Markov chain Monte Carlo chain, we obtained 10,000 thinned posterior samples from 500,000 iterations.

### Associations Between Mobility Proxies and Transmission Dynamics

Time series of the mobility proxies were smoothed by a 7-day rolling average. The Pearson correlation coefficients (*r*) between the mobility proxies and *R_t_*, and between the mobility proxies and *k*, were computed, respectively. The R-square was determined to quantify the proportion of variance explained by a mobility proxy using linear regression. A full regression model was built including all the mobility proxies. *R_t_* and *k* were log-transformed and negative log-transformed in the regression models, respectively. To quantify the relative contribution of a specific mobility proxy in predicting the *R_t_* or *k*, we determined the difference in *R*-squares (ΔR-sq) between a full model and the model without the proxy [31]. We calculated the means and 95% credible intervals (CrIs) for the estimates by sampling 10,000 times from the posterior distributions of the estimates; 3 and 7 days of lags were tested to examine the lag effects of mobility on the outcomes of disease transmissibility ensuring the robustness of the study findings.

The analysis was conducted in R statistical software (version 4.0.3; The R Foundation). The programming code is available upon request.

### Ethics Approval

Ethics approval was obtained from the Survey and Behavioral Research Ethics Committee, The Chinese University of Hong Kong (SBRE-20-581). Because this study was a modeling analysis using secondary data with no personal information, the requirement for obtaining informed consent was waived.

## Results

**Figure 1:**
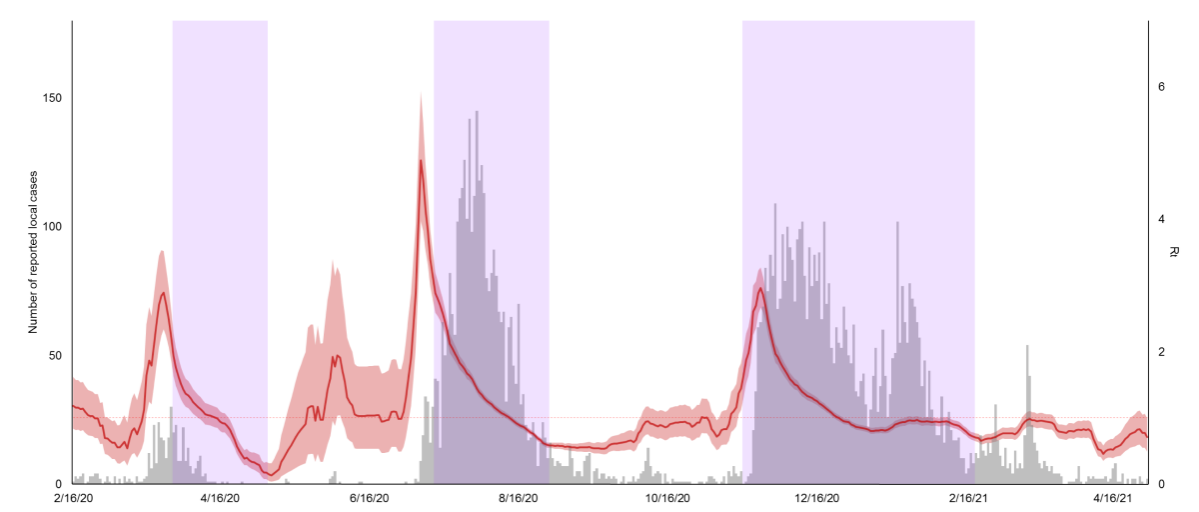
Number of reported cases and estimated time-varying reproduction number (Rt) in Hong Kong from February 16, 2020, to April 30, 2021. The number of reported cases is indicated by the gray bars (left axis), and the posterior median estimate of Rt is indicated by the red line, with shading indicating the 95% credible interval (right axis). The purple shaded areas show the intervention phases at different periods. Phase 1: A table of restaurants was limited to 4 people, and 6 types of premises must close from 6 PM from March 28, 2020, and bars must close from 6 PM from April 3, 2020. The measures started to be relaxed from May 5, 2020 (left); Phase 2: Restaurant dine-in services at night were banned, and no more than 2 persons may be seated together at 1 table in restaurants since July 13, 2020. The measures started to be relaxed since August 28, 2020 (middle). Phase 3: Business hours of restaurants, bars, and clubs were shortened; the number of people allowed to be seated together at 1 table was reduced; and the number of people participating in any 1 banquet in catering premises would be limited to 40 since November 16, 2020. Dine-in services at restaurants from 6 PM to 5 AM of the next day were banned, and the number of people participating in a banquet was further limited to 20 since December 10, 2020. The measures started to be relaxed since February 18, 2021 (right).

**Figure 2:**
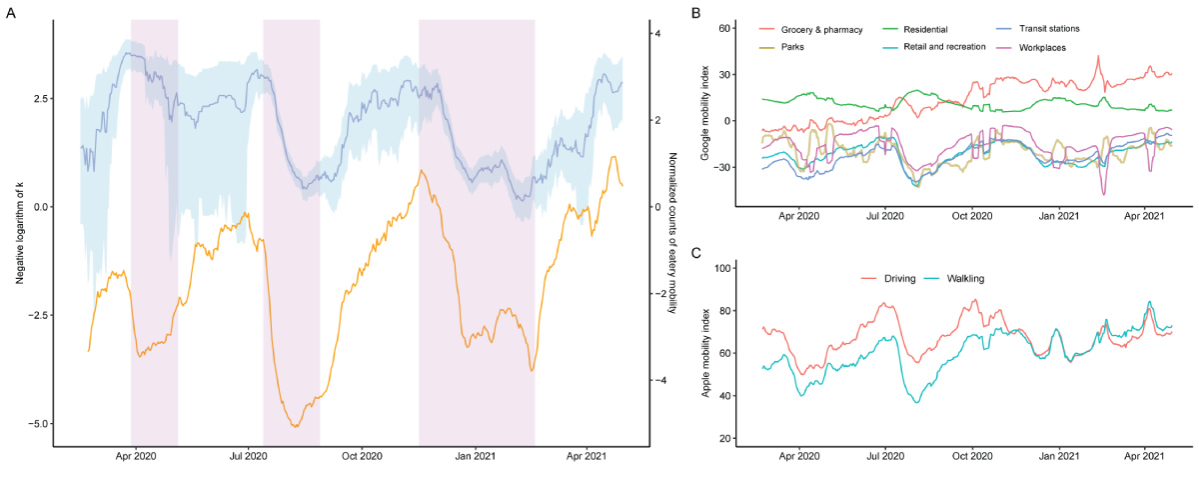
Negative logarithm of dispersion parameter (k) and mobility proxies. A. The negative logarithm of *k* is indicated by the blue line, with shading indicating the 95% credible interval (left axis), and the normalized counts of dining-out mobility is indicated by the orange line (right axis). B. The Google Mobility Index by types. C. The Apple Mobility Index by types.

The relationship between and among social distancing policies, disease transmission dynamics, and the mobility proxies from February 16, 2020, to April 30, 2021, is illustrated in Figure 1 and Figure 2. The estimated *R_t_* decreased from 1.94 (95% CrI 1.63 to 2.29) to 0.17 (95% CrI 0.07 to 0.34), from 2.88 (95% CrI 2.59 to 3.20) to 0.59 (95% CrI 0.55 to 0.63), and from 1.63 (95% CrI 1.29 to 2.01) to 0.72 (95% CrI 0.66 to 0.78) at 3 intervention phases, respectively.

A total of 6391 clusters involving 8375 cases were reported during the study period. Among the identified clusters, there were 4527 clusters with a size equal to 1 (ie, sporadic cases). The distribution of the number of secondary cases generated by the primary cases for the whole study period is showed in Multimedia Appendix 1. Of the 7194 linked cases, 1096 (15.23%) cases generated at least 2 secondary cases, and 1 case even initiated a transmission cluster composing 394 cases. The cluster data were used to estimate *k*, which was negative log-transformed, so the larger the value, the higher the superspreading potential. At the 3 intervention phases, the negative-log *k* decreased from 3.49 (95% CrI 3.12 to 3.83) to 2.34 (95% CrI –0.70 to 3.30), from 3.02 (95% CrI 2.63 to 3.37) to 0.69 (95% CrI 0.28 to 1.06), and from 2.51 (95% CrI 1.61 to 3.17) to 0.68 (95% CrI 0.09 to 1.19), respectively (Figure 2).

Similar decreasing trends were observed for the mobility proxies (Figure 2 and Table 1). A high correlation between dining-out behavior and *k* was observed (*r*=0.46, 95% CrI 0.32 to 0.56). While several other mobility proxies were also highly correlated with *k*, including visiting retail and recreation (*r*=0.41, 95% CrI 0.24 to 0.54), parks (*r*=0.36, 95% CrI 0.17 to 0.51), and workplaces (*r*=0.37, 95% CrI 0.24 to 0.46), mobility proxies of transit stations, driving, and walking had a weaker correlation with *k* (*r*<0.15). Correlations between mobility proxies and *R_t_* were generally weaker. Only the mobility of dining-out behavior in eateries (*r*=0.16, 95% CrI 0.14 to 0.18) and workplace (*r*=0.13, 95% CrI 0.10 to 0.16) maintained a larger correlation with *R_t_*.

**Figure 3:**
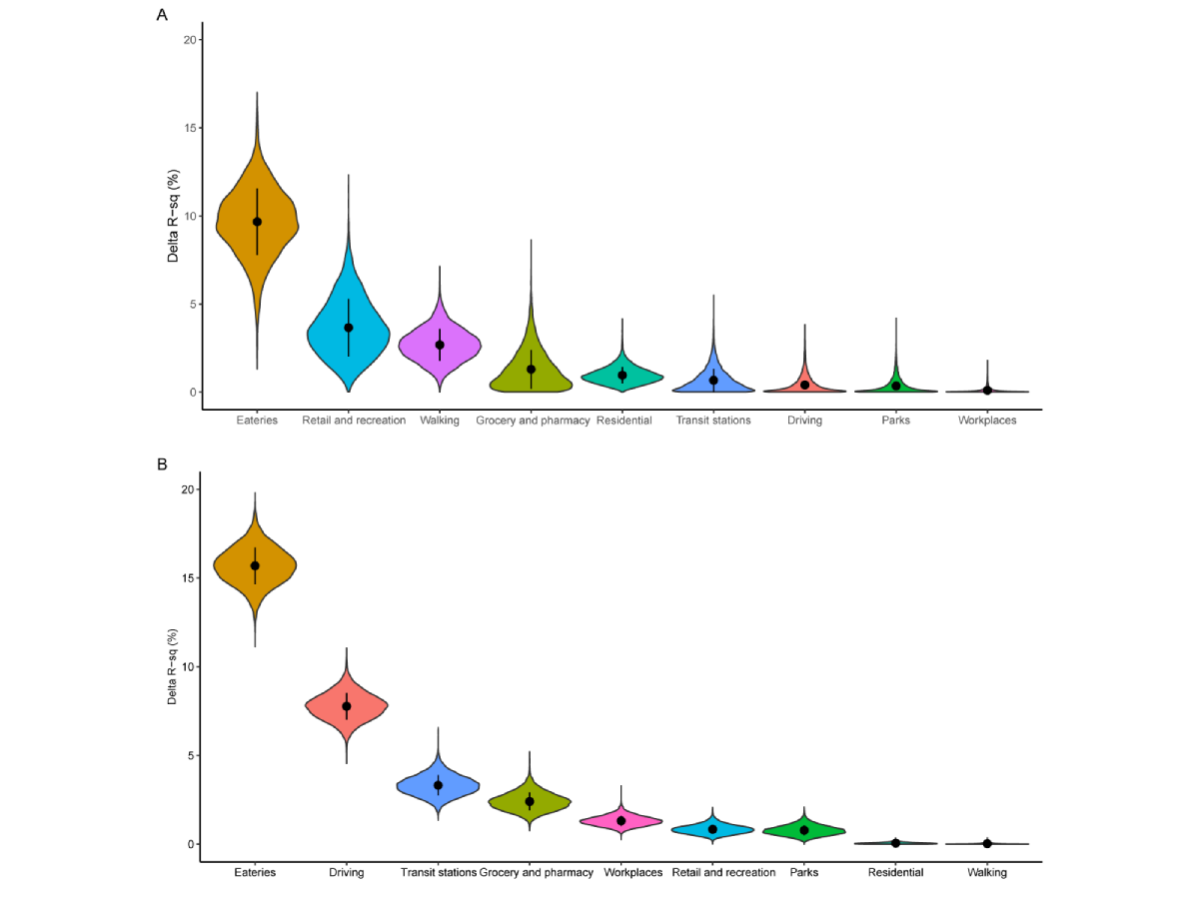
Relative contributions of the mobility proxies in predicting dispersion parameter (k) and time-varying reproduction number (*R*t). A, B: The relative contributions of each of the mobility proxies, including eateries, retail and recreation, grocery and pharmacy, parks, transit stations, workplaces, residential, driving, and walking, in predicting (A) *k* and (B) *R*t were quantified by the differences in *R*-squares (ΔR-sq). The black dot indicates the mean estimate of ΔR-sq interpolated with an SD.

The mobility proxies could explain a higher percent of variability of *k* than that of *R_t_*. In the full model, including all the mobility proxies, the *R*-squares were 54.3% (95% CrI 30.3% to 75.2%) and 23.7% (95% CrI 21.5% to 25.9%) for regressing *k* and *R_t_*, respectively. We used ΔR-sq to quantify how much of the variabilities of the *k* and *R_t_* were explained by each of the mobility proxies (Figure 3 and Table 1). Among the proxies, the mobility of dining-out behavior explained the most of variability of *k* (ΔR-sq=9.7%, 95% CrI 5.7% to 13.2%) and *R_t_* (ΔR-sq=15.7%, 95% CrI 13.6% to 17.7%). Other mobility proxies result in a mean ΔR-sq of less than 5% when regressing *k*. For *R_t_*, the proxies of driving and transit stations had a ΔR-sq of 7.8% (95% CrI 6.3% to 9.3%) and 3.3% (95% CrI 2.3% to 4.5%), respectively. Other mobility proxies had the mean ΔR-sq<3% when regressing *R_t_*.

We examined the effects of 3-day and 7-day lags of mobility proxies on *k* and *R_t_* (Multimedia Appendix 2). In general, dining-out behavior still explained the highest variance of the outcomes. The ΔR-sq of a 3-day lag of the metric was 7.8% (95% CrI 4.2% to 11.4%) and 13.1% (95% CrI 11.1% to 15.2%) for *k* and *R_t_*, respectively, whereas that of a 7-day lag of the metric was 4.1% (95% CrI 2.1% to 6.8%) and 11.7% (95% CrI 9.9% to 13.7%) for *k* and *R_t_*, respectively. The changes in ΔR-sq for other mobility proxies were also not apparent when their lag effects were considered. Apart from that, a change of the assumed serial interval (mean 4.6, SD 4.9 days) only resulted in a minor change in the ΔR-sq for *R_t_* with the primary findings remaining unaffected (Multimedia Appendix 3).

**Table 1:** Associations between mobility proxies and transmission dynamics^a^.

Mobility proxies	*R_t_* ^b^			*k* ^c^	
	*r* ^d^	ΔR-sq^e^ (%)	*r*		ΔR-sq (%)
Dining-out behavior	0.16 (0.14 to 0.18)	15.7 (13.6 to 17.7)	0.46 (0.32 to 0.56)		9.7 (5.7 to 13.2)
Retail and recreation	0.00 (–0.02 to 0.02)	0.8 (0.4 to 1.4)	0.41 (0.24 to 0.54)		3.7 (1.0 to 7.3)
Grocery and pharmacy	–0.01 (–0.04 to 0.03)	2.4 (1.5 to 3.5)	–0.23 (–0.36 to –0.04		1.3 (0.0 to 4.1)
Parks	–0.10 (–0.12 to –0.08)	0.8 (0.3 to 1.4)	0.36 (0.17 to 0.51)		0.4 (0.0 to 1.6)
Transit stations	0.02 (–0.01 to 0.04)	3.3 (2.3 to 4.5)	0.16 (0.06 to 0.24)		0.7 (0.0 to 2.3)
Workplaces	0.13 (0.10 to 0.16)	1.3 (0.8 to 1.9)	0.37 (0.24 to 0.46)		0.1 (0.0 to 0.5)
Residential	–0.03 (–0.05 to –0.01)	0.1 (0.0 to 0.2)	–0.21 (–0.29 to –0.11)		1.0 (0.2 to 2.0)
Driving	0.02 (0.00 to 0.05)	7.8 (6.3 to 9.3)	0.11 (–0.01 to 0.22)		0.4 (0.0 to 1.8)
Walking	–0.05 (–0.08 to –0.03)	0.0 (0.0 to 0.1)	0.01 (–0.08 to 0.08)		2.7 (1.0 to 4.7)

^a^The results were presented using means and 95% credible intervals of the estimates from the posterior distributions. Proxy of dining-out behavior was retrieved from OpenRice. Proxies of retail and recreation, grocery and pharmacy, parks, transit stations, workplaces, and residential were retrieved from Google COVID-19 community mobility reports. Proxies of driving and walking were retrieved from Apple mobility trends.

^b^*R_t_*: time-varying reproduction number.

^c^*k*: dispersion parameter of superspreading potential.

^d^*r*: Pearson correlation coefficient.

^e^ΔR-sq: difference in *R*-squares between a full model and the model without the proxy.

## Discussion

### Principal Findings

Digital data on human mobility play an important role in tracking public compliance with NPI as well as monitoring infectious disease dynamics. While many studies evaluated the reliability of different digital mobility metrics as a proxy of the SARS-CoV-2 transmission [17-21], none have examined the relationship between the trend of dining-out behaviors and the superspreading potential of COVID-19. In this study, we evaluated the reliability of dining-out activities at eateries as a proxy for SARS-CoV-2 transmission risk in Hong Kong with outbreak highly characterized by SSE. According to our results, dining out activities were associated with the change in disease transmissibility as well as the superspreading potential of COVID-19. This finding is consistent with the fact that eatery venues were high-risk settings for frequent virus exposure and superspreading, as they tend to be indoors, populated, and without the mandate of mask wearing, having the highest proportion of COVID-19 infection counts than other places [32,33]. According to the COVID-19 spread pattern in Hong Kong, as characterized by previous research, eateries had a higher outbreak potential of unlinked cases than other settings and accounted for the second highest number of linked transmissions after the households [15,16], as dining-out activities gather individuals that are not socially connected in adjacent and common areas of the restaurant, widening the dispersion of infected persons and subsequently sustaining case clusters propagated across different settings. Similar to our results, the county-level COVID-19 growth in the United States increased from 1.1% to 1.2% when dining-out restrictions were removed, and a 55% decline in new COVID-19 cases was found after imposing bans on indoor on-premises dining [3,4].

During the COVID-19 epidemic, Hong Kong did not impose any stringent measures on eatery settings (eg, compulsory banning of indoor dining and closures of eatery venues), which has been adopted in a number of countries such as the United Kingdom and Singapore, before the availability of COVID-19 vaccines [34,35]. Nevertheless, as demonstrated in this study, less stringent measures such as shortening the business hours and restricting the capacity of eateries were still able to decrease the related mobility as well as the superspreading potential, particularly in the second and the third intervention phases. The finding supports the effectiveness of these measures, even though the related effect on transmission has been questioned [36]. However, we cannot factor in the effects of other social distancing measures implemented, although by including other mobility proxies in our analyses, we have adjusted for some secular effects from the other measures.

Of the mobility proxies, mobile device data including the community mobility reports from Google LLC and mobility trend reports from Apple Inc have been well studied as proxies for changes in human activities and SARS-CoV-2 transmissibility [17-19]. For instance, a Japanese investigation demonstrated that Apple mobility data were reliable for a short-term prediction of COVID-19 transmission [19], whereas an Italian investigation showed the changes in transmissibility of SARS-CoV-2 were associated with both social distancing measures and Google mobility [18]. While the studies assured the reliability of these metrics, we showed that dining-out behavior had a greater contribution to the COVID-19 transmissibility compared with the digital proxies generated by Google and Apple, likely because their data are not specific to eateries. Nevertheless, our analysis showed that the movement metrics including driving and transit stations still accounted for a certain proportion of variability of the community transmission. This observation echoes a previous study in Hong Kong using digital transactions in public transport and social mixing data to forecast the COVID-19 epidemics [20]. Similar movement metrics provided by Baidu Huiyan were also well demonstrated as useful data to improve the temporal and spatial resolution of COVID-19 transmissions [20,21].

In this study, estimating superspreading potential required comprehensive data of rapid contact tracing. In the Omicron epidemic, public health resources for conventional contact tracing were overwhelmed in Hong Kong due to a rapid and huge surge in infections. With insufficient data on case linkages, the superspreading potential cannot be determined during the Omicron period. It suggests a need for novel digital tools for contact tracing, for example, by app-based tools that can notify users instantaneously when their contacts are confirmed positive [37,38]. The deployment of app-based tools at a population level not only improves the timeliness of tracing but also avoids the recall bias in contact tracing. While digital contact tracing is common in many places [39], residents in Hong Kong were skeptical of this type of app due to ethical and privacy concerns [40]. As long as the digital data are available with a large population coverage and adequate compliance, they could be incorporated in our analytical framework that requires comprehensive contact tracing data.

With the comprehensive information of rapid contact tracing before an Omicron outbreak in Hong Kong, the major strength of this study is the capability of using the corresponding data for the estimation of time-varying superspreading potential, which were unlikely to be available in other settings without intense contact tracing during the COVID-19 pandemic. In addition, while a majority of studies related the mobility proxies to reproduction numbers [17,19], our study is the first investigation that linked the mobility pattern to disease superspreading, especially for the COVID-19 transmission, which was found to be highly heterogeneous [12-14,28,41].

### Limitations

Nonetheless, this study had several limitations. First, individual-level data on demographics, duration of indoor dining, and the number of people dining together were not available, thus lowering the resolution of transmission risk imposed on the individual in an eatery. Still, the aggregated information provided a simple, inexpensive, and readily available data source for informing decisions for pandemic control and predicting the superspreading potential. Second, dining-out behavior may be different by geographic locations given the differential distributions of eateries. Due to a lack of the geographic information of the mobility proxies, the modeling analysis was unable to account the spatial variation, and the study findings may thus suffer from bias. Third, our study did not intend to develop a forecast model given limited mobility and social mixing data [20]. We expect that an increased coverage of app-based tools would offer high-resolution data for further development of a prediction model. Fourth, a single data source of proxy for the dining-out behavior was used for this study. While OpenRice was the most common web page for commenting and rating different eateries, we were unable to include diners who preferred using other web page or apps such as Facebook.

### Conclusion

In conclusion, we demonstrated that there was a strong link between dining-out behaviors and the superspreading potential of COVID-19, and the metric was more reliable than other common mobility data derived by Google and Apple Inc. The findings suggest that the mobility proxy of dining-out behavior is able to help health officials for monitoring the public compliance of social distancing measures as well as the cluster outbreak potential. For example, officials could adjust the intensity of social distancing measures (eg, restriction of the maximum number of seats in a restaurant and mandatory closure of eateries) based on the disease transmissibility inferred by the mobility proxies. In addition, our methodological innovation recommends a further development of using digital mobility proxies of dining-out patterns to generate early warnings of SSE, thereby assisting in resource planning on corresponding measures.

## Appendix

Multimedia Appendix 1 The observed offspring distribution with fitted curve for the transmission chains, February 16, 2020, to April 30, 2021. Dotted dark blue curve is the estimated probability density function of the negative binomial distribution.

Multimedia Appendix 2 Relative contributions of (A, B) 3-day and (C, D) 7-day lag effects of mobility proxies in predicting dispersion parameter (k) and time-varying reproduction number (*R*t). The relative contributions of each of the mobility proxies, including eateries, retail and recreation, grocery and pharmacy, parks, transit stations, workplaces, residential, driving, and walking, in predicting (A, C) *k* and (B, D) *R*t were quantified by the differences in *R*-squares (ΔR-sq). The black dot indicates the mean estimate of ΔR-sq interpolated with an SD.

Multimedia Appendix 3 Relative contributions of the mobility proxies in predicting time-varying reproduction number (Rt) when the mean and SD of the assumed serial interval distribution were changed to 4.6 and 4.9 days, respectively. The relative contributions of each of the mobility proxies, including eateries, retail and recreation, grocery and pharmacy, parks, transit stations, workplaces, residential, driving, and walking, in predicting *R*t were quantified by the differences in *R*-squares (ΔR-sq). The black dot indicates the mean estimate of ΔR-sq interpolated with an SD.

## Abbreviations

CrI: credible interval
NPI: nonpharmaceutical interventions
SSE: superspreading events

## Data Availability

The sharing of data is restricted by the Department of Health, Hong Kong.
