# Supplementary figures and images for: Dining-Out Behavior as a Proxy for the Superspreading Potential of SARS-CoV-2 Infections: Modeling Analysis
Source: JMIR Public Health Surveill. 2023 Mar 7;9:e44251. doi: 10.2196/44251 (PMC9994464; doi:10.2196/44251)

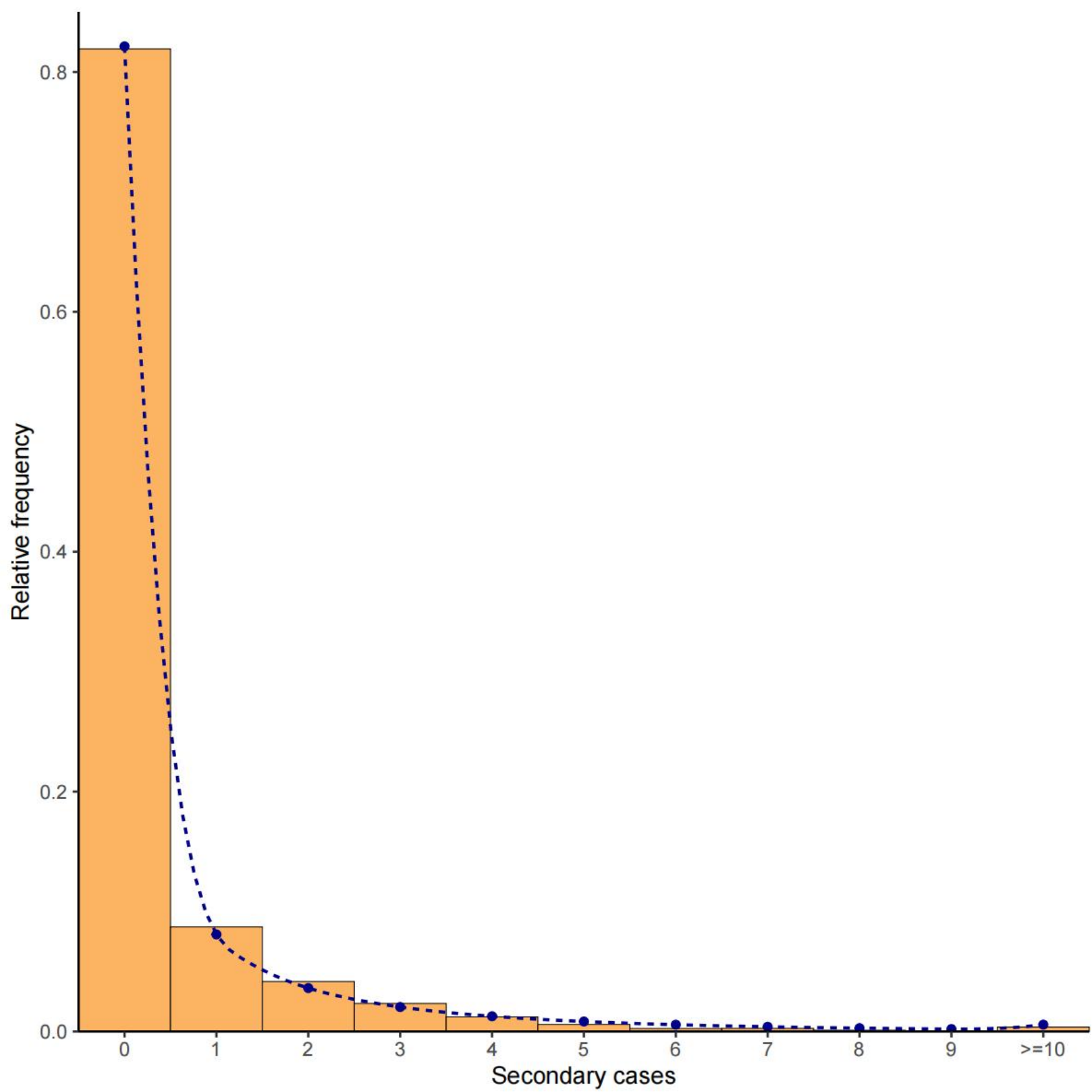

Supplement: Multimedia Appendix 1 [file publichealth_v9i1e44251_app1.pdf]

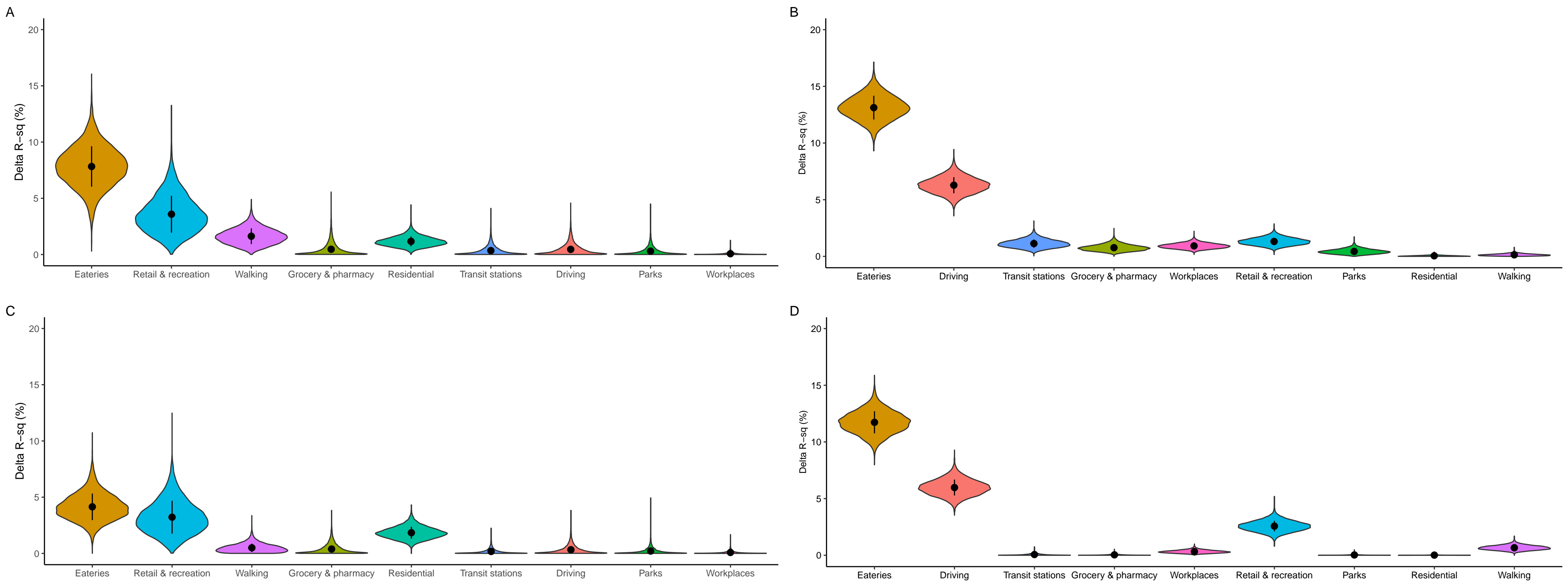

Supplement: Multimedia Appendix 2 [file publichealth_v9i1e44251_app2.pdf]

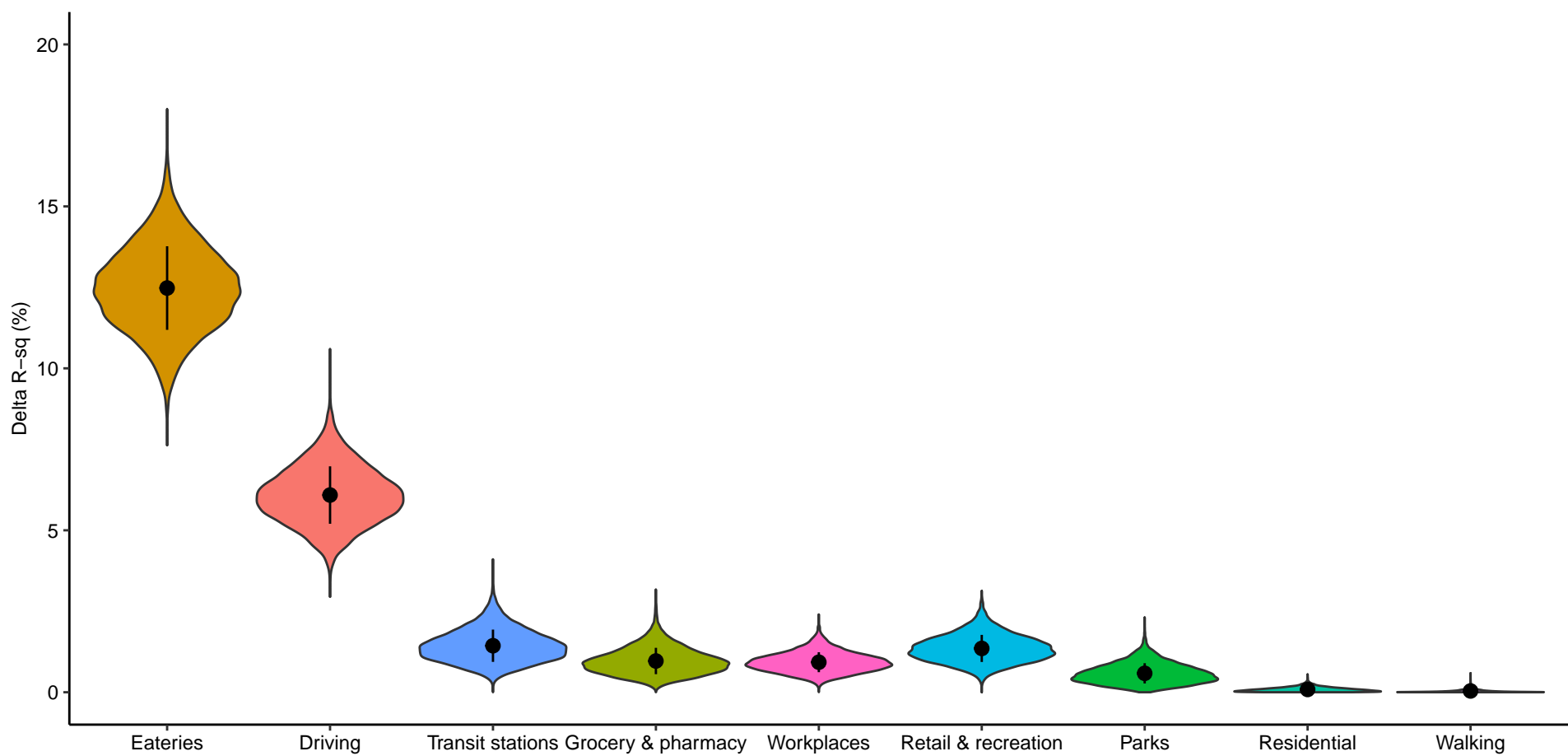

Supplement: Multimedia Appendix 3 [file publichealth_v9i1e44251_app3.pdf]
